# Apocynin combined with drugs as coadjuvant could be employed to prevent and/or treat the chronic kidney disease

**DOI:** 10.1080/0886022X.2017.1421557

**Published:** 2018-01-04

**Authors:** Jorge Osvaldo Montes-Rivera, Feliciano Tamay-Cach, Julio César Quintana-Pérez, Juan Alberto Guevara-Salazar, José Guadalupe Trujillo-Ferrara, Leonardo Del Valle-Mondragón, Mónica Griselda Arellano-Mendoza

**Affiliations:** a Laboratorio de Investigación en Enfermedades Crónico Degenerativas, Sección de Estudios de Posgrado e Investigación, Departamento de Formación Básica Disciplinaria, Escuela Superior de Medicina, Instituto Politécnico Nacional, Ciudad de México, México;; b Sección de Estudios de Posgrado e Investigación, Departamento de Formación Básica Disciplinaria, Escuela Superior de Medicina, Instituto Politécnico Nacional, Ciudad de México, México;; c Departamento de Farmacología, Instituto Nacional de Cardiología “Ignacio Chavéz”, Ciudad de México, México

**Keywords:** Apocynin, chronic renal failure, nephrectomy, endothelial dysfunction, oxidative stress

## Abstract

A worldwide public health problem is chronic kidney disease (CKD) presenting alarming epidemiological data. It currently affects about 10% of the adult population worldwide and has a high mortality rate. It is now known that oxidative stress represents one of the most important mechanisms in its pathophysiology, from the early stages to the terminal phase. Oxidation increases inflammation and reduces the capacity of NO**^•^** to relax vascular smooth muscle, in part by decreasing bioavailability of tetrahydrobiopterin (BH_4_), leading to endothelial dysfunction and high blood pressure, and due to the limited effectiveness of existing treatments, new drugs are needed to prevent and/or treat these mechanisms. The aim of this study was to test apocynin in a 5/6 nephrectomy mouse model of CKD to investigate whether its known antioxidant effect can improve the disease outcome. This effect results from the inhibition of NADPH oxidase and consequently a reduced production of the superoxide anion (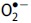). Animals were divided into five groups: sham, 5/6 nephrectomy only, and 5/6 nephrectomy followed by treatment with captopril, losartan or apocynin. The parameters evaluated were blood pressure and markers of oxidative stress (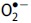) and endothelial function (BH4). There were significantly lower levels of 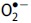 and a greater availability of serum BH4 in the apocynin-treated animals versus the control group and the two other drug treatments. The present findings suggest that apocynin in conjunction with a coadjuvant for modulating blood pressure may be useful for controlling the progression of CRF.

## 1. Introduction

According to the Pan-American Health Organization (PHO) and World Health Organization (WHO), chronic kidney disease (CKD, also known as chronic kidney failure) causes a high percentage of morbidity and mortality. The alarming epidemiological data reveal a serious public health problem currently affecting 10% of the adult population worldwide. The PHO and WHO define CKD as a progressive, silent disease that does not present symptoms until advanced stages, and the treatments are more expensive (e.g., dialysis and hemodialysis and kidney transplantation). Many countries do not have enough specialists or the sufficient resources/equipment to provide treatment for all those suffering from this malady [1].

In adults, CKD is defined as the presence of a renal structural alteration, demonstrable by specialized urine tests (showing urinary sediment) as well as imaging or histopathological studies. This condition persists for a minimum of 3 months and its onset is not always accompanied by impaired renal function [2]. The 2002 and 2004 KDOQI (Kidney Disease Outcomes Quality Initiative) guidelines have classified CKD into five stages, each involving an order of progression and aggravation of the disease that is mainly defined by the glomerular filtration rate (GFR) [3]. The KDIGO (Kidney Disease: Improving Global Outcomes) guidelines of 2014 further subclassified these stages of CDK for greater precision in diagnosis, evaluation, management and prognosis of affected patients [4]. CDK is now properly defined as a decrease in renal function, expressed by the GFR or an estimated creatinine clearance <60 mL/min/1.73 m^2^, corresponding to stages 3a, 3b, 4 and 5 of the disorder (Table 1) [3–5].

**Table 1.** Stages of chronic kidney disease (CKD) modified by the KDIGO 2013.

Stages grade (G)	Glomerular filtration rate (ml/min/1,73 m^2^)	Description
G1	≥ 90	Normal or high
G2	60–89	Slightly decreased
G3a	45–59	Slightly or moderately decreased
G3b	30–44	Moderately or severely diminished
G4	15–29	Severely diminished
G5	<15	Renal failure

Terminal CKD (TCKD) is usually the final outcome of pathologies that affect the kidney in a chronic way. Although alterations in kidney tissue structure, redox balance and endothelial function are reversible in stages 1, 2 and 3, these conditions are irreversible once reaching stages 4 and 5 [2], having deteriorated the antioxidant defenses beyond repair. The complications in these cases are mainly caused by the elevated production of reactive oxygen species (ROS) and reactive nitrogen species (RNS), thus altering the oxidant–antioxidant balance. ROS and RNS are an integral part of metabolism, stress and inflammation [6] and have been extensively studied in disorders of the renal system. Since numerous reports confirm the importance of the oxidative state in renal tissue, antioxidant therapy has become a part of the preventive and regulatory treatment for CKD, delaying its progression (KDOQI 3) and consequently the appearance of the terminal phase (TCKD)[7,8].

Factors that promote oxidative imbalance are a fundamental part of the basic pathophysiological mechanisms of renal disorders. Ischemic or toxic phenomena, which can engender acute damage to the tubule, may be accompanied by excessive production of ROS. In chronic kidney damage of metabolic origin (e.g., diabetes, hypertension and autoimmune diseases), overproduction of ROS is also commonly found [9].

The natural defense system against oxidative stress consists mainly of nonenzymatic antioxidants (e.g., reduced glutathione, peroxiredoxin, vitamin E, vitamin C, ferritin, transferrin, ceruloplasmin and albumin) and antioxidant enzymes, such as superoxide dismutase (SOD), catalase (CAT), thioredoxin reductase and glutathione peroxidase (GPx). All these elements are closely related to the progression of the different stages of CKD [4]. In patients with damaged renal function, excessive levels of ROS and other uremic toxins of an oxidizing nature disrupt the redox balance and overcome the capacity of the antioxidant defense [10].

In CKD, the main source of ROS is NADPH oxidase (Nox), which is a multienzyme complex located in the plasma membrane and in the cytosol of endothelial cells and vascular smooth muscle. The different isoforms of Nox are Nox1, Nox2 and Nox4 [11], the latter showing greater expression in endothelial cells than Nox2 [10]. Under conditions of oxidative stress, however, Nox2 [12] generates eight times more of the superoxide anion (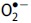), increasing its level 2 to 3 times. Hence, 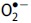 reaches a greater concentration than other isoforms in endothelial cells [13].

When overproduced, 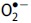 combines with the nitric oxide radical (NO**^•^**) and forms a highly reactive pro-inflammatory compound, the peroxynitrite anion (ONOO^−^). The rise in extracellular ONOO^−^ levels decreases the bioavailability of NO**^•^** by limiting its diffusion to vascular smooth muscle, which in turn diminishes the capacity of blood vessels to relax. At the level of arterioles (interlobular, arcuate, afferent, efferent and rectal vessels), the resulting endothelial dysfunction is characterized by altered regulation of vasodilation and vascular tone, leading to ischemia and finally to renal parenchymal necrosis. In addition, ONOO^−^ interacts with tyrosine residues of the proteins producing 3-nitrotyrosine. By oxidizing tetrahydrobiopterin (BH_4_) and thereby favoring the decoupling of endothelial nitric oxide synthase (eNOS), ONOO^−^ triggers even greater vascular 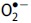 production and, consequently, tissue damage [14].

Angiotensin II (Ang II) also modifies redox balance by stimulating Nox in a reaction that mainly yields 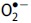, which in turn oxidizes NO**^•^**. Once again, this reduces the capacity of NO**^•^**to relax vascular smooth muscle and regulate vascular tone, resulting in elevated vascular resistance [15]. Ang II is implicated in the development of various cardiovascular and renal pathologies. Apart from being synthesized by the renin–angiotensin–aldosterone system (RAAS), it is produced by alternate enzymatic pathways mediated by cathepsin G, chymase, chymostatin-sensitive angiotensin-generating enzyme (CAGE) and tissue plasminogen activator (T-PA). Hence, each of these pathways can indirectly contribute to increased vascular tone [16].

On the other hand, 4-hydroxy-3-methoxyacetophenone (apocynin), isolated from the medicinal plant *Picrorhiza kurroa*, has gained recognition as an inhibitor of Nox. Although the mechanism of action is not yet completely clear, it is known that apocynin inhibits assembly of the cytoplasmic subunits of Nox: p47phox, p67phox and p21rac with gp91phox and p22phox of membranes [17,18]. Apocynin binds to p47phox in the same region in which the latter protein binds to p22phox. With the inhibition of Nox, there is a lower level of 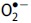 and an enhanced bioavailability of NO**^•^** [11,19].

Due to its effects on redox balance and vascular tone, the Nox enzyme has become an important therapeutic target. The aim of the present study was to evaluate the effects of apocynin as a Nox inhibitor in a 5/6 nephrectomy mouse model (with C57BL/6 mice) of CRF. The probable decrease in 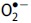 production was explored as well as its effect on the pathophysiology of CKD. This investigation is part of the current effort to test new inhibitors of Nox for their possible renoprotective effect. The long-term aim is to improve the quality of life of patients while reducing the morbidity and mortality of CDK. The antioxidant approach to treating kidney disease is in accordance with guidelines of the Kidney Dialysis Outcomes Quality Initiative (KDOQI 3).

## 2. Materials and methods

### 2.1. Animals and treatments

The study was carried out with 30 C-57BL/6 mice, which were treated according to guidelines for the care and use of laboratory animals issued by the Sección de Estudios de Posgrado e Investigación of the Escuela Superior de Medicina, the Instituto Politécnico Nacional [20], and the Official Mexican Norm (Norma Oficial Mexicana, NOM-062-ZOO-1999) [21]. The cadavers and biological waste of the mice were considered as biologically infectious hazardous waste and therefore handled according to the Official Mexican Norm (Norma Oficial Mexicana, NOM-087-ECOL-SSA1–2002) [22].

The animals were divided into five groups (*n* = 6), with a false (sham) surgery, a 5/6 nephrectomy only, and a 5/6 nephrectomy followed by oral treatment at 10 mg/kg/day with one of three drugs for 14 days. Two of the drugs, captopril and losartan, are currently employed in clinical practice, while the test drug was apocynin.

### 2.2. Nephrectomy

As reported by Jing et al., a 5/6 nephrectomy was used to reproduce chronic renal failure in a C57BL/6 mouse model. Briefly, the 5/6 nephrectomy was performed after intraperitoneally (i.p.) injecting sodium pentobarbital (60 mg/kg body weight) as anesthesia. A left paravertebral cut was made in the dorsal region of the mouse with a scalpel, dissecting the skin, muscle and adipose tissue to allow for the localization of the left kidney at the retroperitoneal level. The parietal and visceral peritoneum was dissected and the left kidney was momentarily exposed. Utilizing an optical microscope with a 2× lens, 2/3 branches of the left renal artery were ligated with 4–0 silk thread (partial left nephrectomy). At the right paravertebral level, the retroperitoneum was dissected to reach the hilum of the right kidney, where the renal vessels and renal pelvis (renal hilum) were ligated with silk thread 4–0 to remove the kidney (total right nephrectomy). The retroperitoneum and muscle were then sutured with 4–0 silk thread and the skin with 4–0 nylon. The animals were returned to their individual cages. For the group of sham mice, only the paravertebral and retroperitoneal incisions (without the 5/6 nephrectomy) were made, and the cut was closed as aforementioned, thus promoting similar stress as a 5/6 nephrectomy [23]. After 14 days of drug treatment (or no treatment), the mice were sacrificed with a lethal dose of sodium pentobarbital (30–40 mg/kg) administered i.p., in accordance with Official Mexican Norm (Norma Oficial Mexicana, NOM-062-ZOO-1999) [21].

### Determining the concentration of the superoxide anion (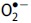)

2.3.


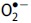
 was quantified by the dihydroethidium (DHE) technique described by Mahfouz et al. at 14 days postnephrectomy. Briefly, one-third of vital renal tissue was used, being the left renal tissue in the case of the 5/6 nephrectomy and sham groups. A portion of the left kidney (approximately 2/3) from renal necrotic tissue was observed macroscopically, as was a portion (approximately 1/3) of vital renal tissue, withdrawing the necrotic portion with a scalpel blade. Subsequently, the third of viable renal tissue was placed on a cubic support (made of aluminum foil), oriented in a transverse direction and completely covered with a freezing gel O.C.T. (ELECTRON MICROSCOPY SCIENCES No. Catalog 4583 [Tissue-Tek]), then placed in liquid nitrogen for 60–70 s, allowing for 10 μm cuts to be made on a microtome at −20 °C. The tissue slices were placed on poly-L-Lysine covered slides and incubated at 37 °C for 60 min in the presence or absence of 120 U/mL polyethylene glycol-superoxide dismutase (PEG-SOD, Sigma-Aldrich St. Louis, MO). Afterwards, 10 μM DHE (Molecular Probes D1168, Eugene, OR, USA) was added and the samples were left at 37 °C for 30 min, protected from light. Upon completion of this time, tissues were washed three times (10 min each) with PBS and then covered with Vectashield H-1000 (a fluorescence protector) and a resin in order to view the specimens using confocal microscopy (Olympus America INC.FV-300) [24].

### 2.4. Determining the levels of tetrahydrobiopterin (BH_4_)

At day 14 postnephrectomy, blood samples were extracted by direct cardiac puncture, utilizing a 1 mL syringe with a 25 G × 16 mm needle. Samples were placed in 2 mL aliquots, centrifuged for separation of the serum and globular package. The serum was separated with Sali pipettes in aliquots to make the determination of BH_4_. As described by Baker et al. [25], capillary electrophoresis was employed to detect a fluorescent zone created by laser excitation (equipment: P/ACE™ MDQ Capillary Electrophoresis System, Beckman Coulter).

### 2.5. Measuring systolic blood pressure (SBP)

At 14 days postnephrectomy, the SBP was assessed using the technique reported by Gava et al. Mice were anesthetized by i.p. administration of 60 mg/kg body weight of sodium pentobarbital. The left carotid artery was channeled with a polyethylene cannula (I.D 0.011 ‘O.D. 0.024’ Clay Adams, New Jersey, NJ) for the measurement of SBP. The other end of the cannula was connected to a complete BLPR WPI transducer linked to DUO-18 software for the acquisition of data, which were recorded for 15 min under stable conditions. Mice were considered hypertensive when the SBP was greater than or equal to 30–40 mmHg compared to the control group (sham) [26].

### 2.6. Statistics

All data are expressed as the mean ± standard error of the mean (SEM). For normally distributed data, statistical significance was evaluated by one-way analysis of variance (ANOVA), followed by *post hoc* and Tukey’s test. Statistical significance was considered at *p* < .05.

## 3. Results

### 3.1. The renal superoxide anion

**Figure 1.**
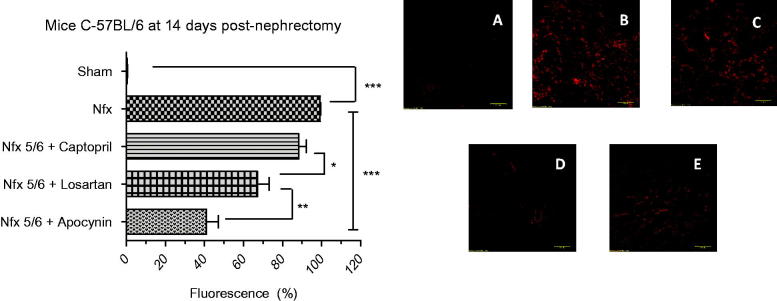
*In situ* evaluation of superoxide anion (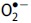) in mouse renal cortex tissue at 14 days postnephrectomy. The percentage (%) of red fluorescence emitted by dihydroethidium, a marker of 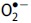 and therefore an indicator of oxidative stress, is illustrated for the different groups of C-57BL6 mice: Sham (image A), a 5/6 nephrectomy without drug treatment (Nfx, image B), and a 5/6 nephrectomy followed by treatment with captopril (Nfx 5/6 + Captopril, image C), losartan (Nfx + Losartan, image D), or apocynin (Nfx + Apocynin, image E). The greatest reduction in the level of 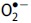 was found with apocynin. Each bar represents the mean ± standard error and statistical significance (***p < .001, **p < .01, *p < .05) was evaluated by one-way analysis of variance (ANOVA), followed by *post hoc* and Tukey’s test.

Upon examining 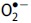
*in situ*, the greatest fluorescence was observed in the 5/6 nephrectomy-only group. There was an 88.22% ± 4.03 decrease in fluorescence emission in the captopril-treated group, 67.11% ± 5.98 in the losartan-treated group and 41% ± 6.11 in the apocynin-treated group (Figure 1). The differences between these groups and the animals receiving a 5/6 nephrectomy only were 11.0% (nonsignificant), 32.11% (*p* < .01) and 58.22% (*p* < .001), respectively, indicating that the most effective drug for reducing oxidation was apocynin.

### 3.2. Serum tetrahydrobiopterin (BH_4_)

**Figure 2.**
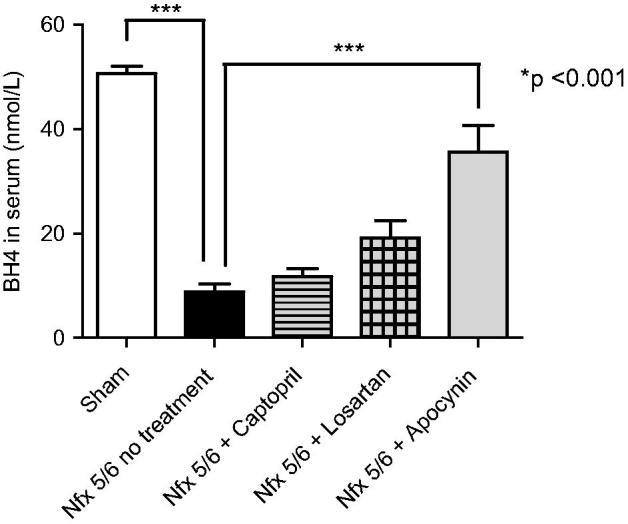
Determination of serum tetrahydrobiopterin (BH_4_) at 14 days postnephrectomy in C57BL6 mice. Illustration of the quantity of BH_4_ (nmol/L), an indicator of the functionality of endothelium tissue, in the serum of the different groups of C-57BL6 mice: Sham, a 5/6 nephrectomy only (Nfx 5/6 no treatment), and a 5/6 nephrectomy followed by treatment with captopril (Nfx 5/6 + Captopril), losartan (Nfx + Losartan) or apocynin (Nfx + Apocynin). Only the apocynin-treated group showed an increased bioavailability of BH_4_. Each bar represents the mean ± standard error and statistical significance (***p < .001) was evaluated by one-way analysis of variance (ANOVA), followed by *post hoc* and Tukey’s test.

Compared to the sham group, the concentration of serum BH_4_ dropped in the animals with a 5/6 nephrectomy only (50.55 nmol/L ± 1.51 vs 8.88 nmol/L ± 1.47), with a difference of 41.67 nmol/L (*p* < .001). In the 5/6 nephrectomy groups receiving drug treatment, a restoration of BH_4_ occurred, finding 11.77 nmol/L ± 1.46 in the captopril-treated group, 19.11 nmol/L ± 3.35 in the losartan-treated group and 35.55 nmol/L ± 5.16 in the apocynin-treated group (Figure 2). However, the only significant difference was in the latter case (26.67 nmol/L; *p* < .001), pointing to apocynin as the most effective drug in this regard.

### 3.3. Systolic blood pressure

**Figure 3.**
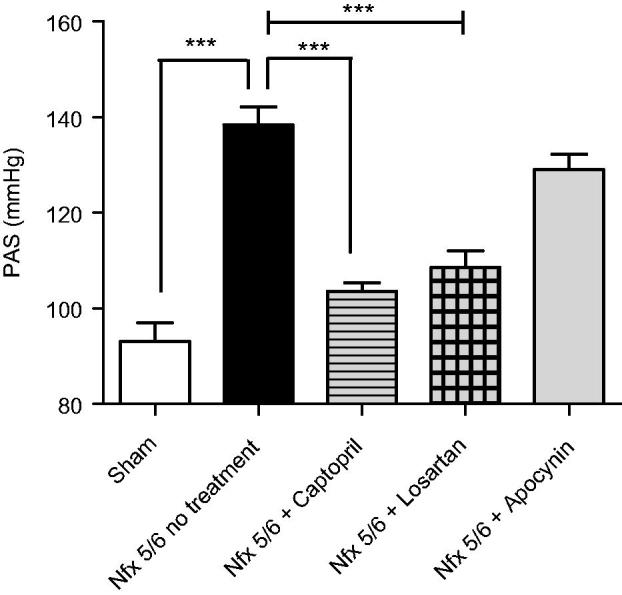
Measurement of the systolic blood pressure (SBP [mmHg]) at 14 days postnephrectomy. The systolic blood pressure (SBP) is shown for the different groups of C-57BL6 mice: Sham, a 5/6 nephrectomy only (Nfx 5/6 no treatment), and a 5/6 nephrectomy followed by treatment with captopril (Nfx 5/6 + Captopril), losartan (Nfx + Losartan), or apocynin (Nfx + Apocynin). The 5/6 nephrectomy caused a sharp increase in the SBP, which was partially attenuated by captopril and losartan. Compared to the mice receiving a 5/6 nephrectomy only, the apocynin-treated group did not present a significant difference in SBP, indicating only a tendency to modulation of this parameter. Each bar represents the mean ± standard error and statistical significance (***p < .001) was evaluated by one-way analysis of variance (ANOVA), followed by *post hoc* and Tukey’s test.

Compared to the sham group, systolic blood pressure (SBP) increased in the animals that underwent a 5/6 nephrectomy only (138.4 ± 3.75 vs 93 ± 3.95 mmHg), with a difference of 45.44 (*p* < .001). Compared to the 5/6 nephrectomy-only group, two of the drug treatments caused the expected decrease in blood pressure: captopril (103.5 mmHg ± 1.84) and losartan (108.6 mmHg ± 3.35), with a difference of 34.9 (*p* < .001) and 29.8 mmHg (*p* < .001), respectively. The apocynin-treated group (129 mmhg ± 3.31) showed a slight decline of 9.4 mmHg in blood pressure, which was not statistically significant (Figure 3).

## 4. Discussion

The efficacy of apocynin in modulating the progression of CKD was compared to that of two drugs in clinical use; captopril and losartan. For this purpose, a 5/6 nephrectomy was performed to establish the conditions of CRF in a mouse model. The parameters measured were 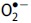 in renal tissue (a marker of oxidative stress) and BH_4_ in serum (an indicator of endothelial function), as well as blood pressure. These three parameters are considered to be altered in the progression of the CKD. In addition, the effectiveness of apocynin was compared to that of captopril and losartan (as representative drugs for their groups). These latter two drugs in clinical use serve as an inhibitor of the angiotensin-converting enzyme (ACE) and Ang II receptor antagonist (ARA II), respectively.

By using microscopy with DHE staining, the greatest quantity of 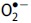 was detected in the renal tissue cortex remnant from the group with a 5/6 nephrectomy only. Consequently, Nox is likely to be the principal source of 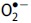, stimulated mainly by Ang II [25] in the model of 5/6 nephrectomy in C57BL/6 mice (Figure 1). This trend was not observed in the sham group. Betancourt et al. found a similar pattern in a CRF biomodel (nephrectomy vs sham), with a significant decline in the activity of the antioxidant enzyme superoxide dismutase (SOD) (0.82 vs 1.14 U/mL/min, respectively) and the levels of reduced glutathione (GSH) (3503.70 vs 4663.10 μg/mL, respectively) [25], a nonenzymatic antioxidant agent at the renal level [25]. The data suggest that Ang II influences the production of 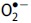 by the direct activation of Nox through its AT1 receptor, as well as by the inherent participation of this enzyme. Hence, 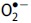 levels were diminished by the treatment with losartan and apocynin, the latter showing the best results. This apparently owes itself to their inhibitory activity of apocynin on the assembly of Nox subunits [10]. On the other hand, captopril did not display efficacy in lowering the levels of 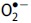, since the production of Ang II is not completely limited by inhibiting ACE. Serna et al., characterized alternate routes of Ang II production, where ACE as well as other enzymes such as chymase, cathepsin G, CAGE and t-PA [16] transform Ang I into Ang II. Consequently, there is a latent capacity for the generation of 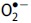 by stimulation of Nox [27].

Regarding serum concentration of BH_4_, significantly lower levels were present in the 5/6 nephrectomy-only animals compared to the sham group (Figure 2). The interaction of 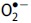 with BH_4_ apparently caused the oxidation of the latter, which would diminish its bioavailability for the coupling and enzymatic activity of endothelial nitric oxide synthase (eNOS). This should have reduced the conversion of L-arginine to L-citrulline and the radical of nitric oxide (NO**^•^**), suggesting a decrease in the production of the latter and a diversion of the reaction into a higher yield of 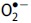. The current findings concur with those reported by Arellano et al., who described that excess ROS can react rapidly to deactivate NO**^•^** and alter the performance of nitric oxide synthase [28]. Additionally, the oxidation of BH_4_ results in the decoupling of eNOS, meaning that the monomers generate 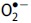 instead of NO**^•^** [29]. Hence, this condition leads to endothelial dysfunction, a characteristic mechanism of CKD (KDOQI 3).

The bioavailability of BH_4_ was enhanced by the three drug treatments herein employed after the 5/6 nephrectomy. Compared to captopril and losartan, apocynin demonstrated a superior effectiveness in this respect. Inhibition of the subunits of Nox engendered a reduced level of 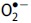 and less oxidation of BH_4_. Thus, the activity of the latter increased and could modulate NO**^•^** levels and restore eNOS coupling and functioning, which implies greater vascular reactivity and the avoidance of endothelial dysfunction [18]. Consequently, the progression of renal damage is delayed and the complications and mortality of CKD are diminished.

Compared to the sham group, a sharp rise was observed in the SBP [mmHg]) of mice with a 5/6 nephrectomy only (Figure 3), corroborating the model as representative of the progression of CKD. The cause of high blood pressure was a greater peripheral vascular resistance, conditioned by a decrease in the area of glomerular filtration, mesangial proliferation, tubulointerstitial inflammation and glomerulosclerosis (renal remodeling), in addition to the production of ROS stimulated by Ang II at the AT1 receptor upon the activation of Nox. On the other hand, Ang II also activates its AT1 receptor at the vascular level, exhibiting independent activity in the production of ROS and consequently the modulation of vascular tone. There was no significant difference in SBP levels between mice in the 5/6 nephrectomy-only group and those treated with apocynin. With apocynin treatment, the slight downward trend in the 5/6 nephrectomy-induced rise in SBP suggests that this parameter is not related solely to the elevated ROS production triggered by the Ang II-induced activation of Nox. As previously mentioned, high blood pressure seems to result from other mechanisms as well, such as renal remodeling and Ang II activity itself, both of which participate in increased vascular resistance [30,31].

In the 5/6 nephrectomy group with drug treatment, captopril and losartan displayed antihypertensive activity by blocking the effects of Ang II, as mentioned by Feldstein et al., where ACE and ARA II achieve a significant blockade of the RAAS. Consequently, there is a reversal of the remodeling generated by profibrotic and inflammatory processes at the vascular and renal levels, which in turn has the beneficial hemodynamic effect of regulating blood pressure in the inferior renal capsule (IRC) [31]. However, the lack of effect of apocynin on blood pressure could possibly be overcome by administering it with a coadjuvant for the treatment of CKD.

## 5. Conclusions

The current findings indicate that apocynin has advantages over losartan and captopril; drugs now used clinically to treat CKD. It seems to better counteract the production of the 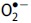 and endothelial dysfunction. Unlike losartan and captopril, on the other hand, apocynin did not show the ability to reduce the high blood pressure characteristic of CKD. Hence, the combination of apocynin with an antihypertensive drug acting as an adjuvant could possibly be employed to treat CKD and reach the therapeutic aim through synergism.
